# The Effect on Intracytoplasmic Sperm Injection Outcome of Genotype, Male Germ Cell Stage and Freeze-Thawing in Mice

**DOI:** 10.1371/journal.pone.0011062

**Published:** 2010-06-11

**Authors:** Narumi Ogonuki, Manami Mori, Akie Shinmen, Kimiko Inoue, Keiji Mochida, Akihiko Ohta, Atsuo Ogura

**Affiliations:** 1 RIKEN BioResouce Center, Tsukuba, Ibaraki, Japan; 2 Department of Life Science, School of Agriculture, Meiji University, Kawasaki, Japan; 3 Graduate School of Life and Environmental Science, University of Tsukuba, Tsukuba, Ibaraki, Japan; 4 Center for Disease Biology and Integrative Medicine, Faculty of Medicine, University of Tokyo, Bunkyo-ku, Tokyo, Japan; Ottawa Hospital Research Institute and University of Ottawa, Canada

## Abstract

**Background:**

Intracytoplasmic sperm injection (ICSI) has been widely used to study the mechanisms of mammalian fertilization and to rescue male-factor infertility in humans and animals. However, very few systematic analyses have been conducted to define factors affecting the efficiency of ICSI. In this study, we undertook a large-scale series of ICSI experiments in mice to define the factors that might affect outcomes.

**Methodology/Principal Findings:**

We used a 5×3×2 factorial design with the following factors: mouse genotype (ICR, C57BL/6, DBA/2, C3H/He, and 129/Sv strains), type of male germ cells (epididymal sperm, elongated or round spermatids), and their freeze–thawing treatment. The efficiencies (parameters) of each developmental step were analyzed by three-way ANOVA (significance level *P*<0.01). The type of male germ cells affected all the four parameters observed: oocyte survival after injection, cleavage of oocytes, implantation, and birth of offspring. Genotype affected the oocyte survival, cleavage and birth rates, whereas freeze–thawing had no effects on any of the parameters. There were significant genotype/cell type interactions for oocyte survival and cleavage, indicating that they were determined by a combination of strain and germ cell maturity. Multiple comparisons revealed that spermatozoa and elongated spermatids gave better implantation and birth rates than did round spermatids, while spermatozoa and elongated spermatozoa were indistinguishable in their ability to support embryonic development. The best overall efficiency (birth rate per oocytes injected) was obtained with frozen–thawed DBA/2 strain elongated spermatids (23.2±4.2%).

**Conclusions/Significance:**

The present study provides the first comprehensive information on ICSI using the mouse as a model and will contribute to the efficient use of materials, time, and efforts in biomedical research and clinics involving ICSI.

## Introduction

Since the first series of reports on successful sperm injection in hamsters in the 1970s [1], [2], intracytoplasmic sperm injection (ICSI, or microinsemination as a broad term) has been widely used to study the mechanisms of mammalian fertilization and to rescue male-factor infertility in humans and animals (for reviews, see [3], [4]). Thanks to improvements in gamete/embryo handling techniques and micromanipulation devices, microinsemination can now be performed using immature spermatogenic cells, including spermatids and spermatocytes to produce normal offspring [5]–[8]. In the mouse, pups can also be obtained using round spermatids from males aged from 17 days [9] to 1000 days [10]. Elongated spermatid injection (ELSI) or round spermatid injection (ROSI) is also becoming very common in other laboratory species, including the rat, mastomys, and rabbit [11]–[14]. As far as we know, normal offspring have been born using spermatozoa or spermatids from 14 animal species [4], but the efficiency varies greatly with the species and the type of male germ cell used. For example, in rabbits no or very few pups were born following ROSI because of a high incidence of aneuploid embryos [13], [14]. This might be caused by the inability of rabbit round spermatids to form a microtubule-organizing center (MTOC) in the ooplasm [15]. In mice, by contrast, the MTOC is formed by the ooplasmic asters so ROSI does not always cause aneuploidy [16], [17], although some aberrant DNA methylation and histone acetylation of the paternal genome has been reported [18], [19]. This unique feature of the mouse zygote has permitted microinsemination to be applied extensively to the production of offspring or embryos with specific genetic or epigenetic characters of interest [20]–[23].

In laboratory mice, reproductive technologies have been developed for the efficient archiving of particular strains in a cryorepository or for safe propagation of their mutant genes. Therefore, many studies have been conducted to reveal strain-dependent effectiveness of the reproductive technologies including superovulation [24]–[26], in vitro fertilization (IVF) [24], [25], [27], and cryopreservation of embryos and gametes [28], [29]. Although ICSI in mice is a relatively new technique, it has already been widely employed in many laboratories because of its consistent fecundity, even in the case of inadequate sperm cryopreservation [30], whole body freezing [31], or failure in spermiogenesis [32]. However, information on the efficiency of ICSI in different strains was very limited except for C57BL/6 and BALB/c [33], [34].

The present study was undertaken to see what biological or technical factors may affect the outcome of microinsemination using the mouse as a model. For statistical analysis by ANOVA, we constructed a complete 5×3×2 factorial design with three factors; i.e., the genotype, the maturity of male gametes, and their freezing treatment. We expect that our large-scale microinsemination experiment data based on statistical analysis would contribute to biomedical research in which ICSI plays important roles for their purposes and goals.

## Materials and Methods

### Animals

C57BL/6NCrSlc and DBA/2CrSlc strain mice (7–10 weeks old) were purchased from Japan SLC (Shizuoka, Japan), and Jcl:ICR, C3H/HeJJcl, and 129*^+Ter^*/SvJcl mice (7–10 weeks old) were purchased from CLEA Japan (Tokyo, Japan). All animal experiments were reviewed and approved by the Animal Experimental Committee at the RIKEN Tsukuba Institute (no. 09-005).

### Collection of Oocytes

Each female mouse was injected intraperitoneally with 7.5 units of equine chorionic gonadotropin (Teikoku-Zoki Pharmaceuticals, Tokyo, Japan), followed by an injection of 7.5 units of human chorionic gonadotropin (hCG; Aska Pharmaceuticals, Tokyo, Japan) 48–50 h later. Mature oocytes were collected from oviducts at 15–17 h after hCG injection and were freed from cumulus cells by a 1 min treatment with 0.1% hyaluronidase (Sigma-Aldrich, St. Louis, MO, U.S.A.) in CZB medium [35]. The oocytes were transferred to fresh CZB medium and incubated at 37°C in an atmosphere of 5% CO_2_ in air until use for microinjection.

### Collection of Spermatozoa and Spermatogenic Cells

To obtain epididymal spermatozoa, the cauda epididymides were removed from 7–10 wk-old male mice and the epididymal contents were dispersed in CZB medium. The spermatozoa were incubated at 37°C in an atmosphere of 5% CO_2_ in air until fresh sperm microinjection (ICSI). The remaining spermatozoa were cryopreserved for later use in frozen/thawed sperm ICSI as described below.

The spermatogenic cells (elongated and round spermatids) were isolated mechanically as described [36]. Briefly, the testes were removed from 7–10 week-old males and placed in erythrocyte lysing buffer (155 mM NH_4_Cl, 10 mM KHCO_3_, 2 mM EDTA, pH 7.2). The seminiferous tubule masses were gently loosened using fine forceps and were washed in cold (5–10°C) Dulbecco's phosphate buffered saline supplemented with 5.6 mM glucose, 5.4 mM sodium lactate, and 0.1 mg/ml of polyvinyl alcohol (polyvinylpyrrolidone, PVP, in the original report) (GL-PBS). The tubules were cut into small pieces and pipetted gently to disperse spermatogenic cells into the GL-PBS. The cell suspensions were filtered through a 38 µm nylon mesh and washed three times by centrifugation (200×*g* for 4 min) in GL-PBS. After being resuspended into GL-PBS, they were immediately used for fresh spermatogenic cell microinjection. The remaining cell suspensions were used for cryopreservation, as described below.

### Cryopreservation and Thawing of Spermatozoa and Spermatogenic Cells

Spermatozoa in CZB medium or spermatogenic cells in GL-PBS were centrifuged (200×*g* for 4 min) and supernatants were removed. They were resuspended in 500 µl of freezing solution (GL-PBS containing 7.5% glycerol and 7.5% fetal calf serum) [37] and transferred into cryotubes (Sumitomo Bakelite, Tokyo, Japan). These tubes were frozen in a deep freezer (−80°C) using a freezing container (Bicell; Nihon Freezer Co. Ltd., Tokyo, Japan) and stored until use. On the day of an experiment, the frozen cryotubes were warmed with tap water for 1 min and added to 1 ml of GL-PBS. They were transferred to centrifuge tubes (15 ml, Iwaki, Tokyo, Japan) with 5 ml of GL-PBS and washed three times by centrifugation (200×*g* for 4 min at 5°C) with GL-PBS. The thawed spermatozoa and spermatogenic cells were resuspended in 100 µl of GL-PBS and used for microinjection.

### Microinjection

Combinations of oocytes and male germ cells from the same strains of mice (ICR, C57BL/6, DBA/2, C3H/He, and 129/Sv strains) were used for microinjection. Intracytoplasmic injection of spermatozoa and spermatids was performed using a micropipette attached to a Piezo-electric actuator (PrimeTech, Ibaraki, Japan), as described [6], [31], [38]. The cover of a plastic dish (50×3 mm; Falcon no. 1006; Becton Dickinson, Franklin Lakes, NJ, USA) was used as a microinjection chamber. Several small drops of Hepes-buffered CZB with or without 12% PVP were placed on the bottom and covered with mineral oil. Spermatozoa and spermatogenic cells were placed in one or two of the Hepes-CZB–PVP droplets. A single spermatozoa was sucked, tail first, into an injection pipette and the head was separated from the tail by applying a few Piezo pulses to the head–tail junction. The isolated sperm head was injected into an oocyte in Hepes-CZB. Elongated and round spermatids were selected morphologically by using a micropipette. Isolated elongated spermatids were each injected into an oocyte. Before injection of the nuclei of round spermatids, oocytes were activated by treatment with Ca^2+^-free CZB medium containing 2.5–5.0 mM SrCl_2_ for 20 min at 37°C. The oocytes were each injected with a round spermatid after activation (at telophase II). The injected oocytes were then kept in Hepes-CZB at room temperature (25°C) for 10 min before being cultured in CZB at 37°C under 5% CO_2_ in air.

### Embryo Culture and Transfer

Embryos that reached the 2-cell stage by 24 h of culture in CZB were transferred into the oviducts (7–10 embryos each side) of pseudopregnant ICR females on the day after sterile mating with a vasectomized male (day 0.5). On day 19.5, the recipient mice were killed and their uteri were examined for the presence of live term fetuses.

### Statistical Analysis

Each experiment was replicated at least three times. We analyzed the effects of three treatment factors (genotype, type of male germ cells, and their freezing treatment) on developmental parameters that reflected the efficiency of microinsemination: 1) the proportion of oocytes that survived after microinjection; 2) the proportion of oocytes that cleaved into 2-cell embryos (per surviving oocytes); 3) the proportion of implanted embryos (per embryos transferred); 4) the birth rate (per embryos transferred); and 5) overall efficiency (proportion of offspring per oocytes injected). All developmental parameters, calculated as percentages, were transformed using arcsine transformation and then analyzed by three-way analysis of variance (ANOVA) using the SPSS program (SPSS Inc., Chicago, IL, USA). The Tukey–Kramer procedure was used for multiple comparisons. A probability of *P*<0.01 was considered statistically significant.

## Results

**Figure 1:**
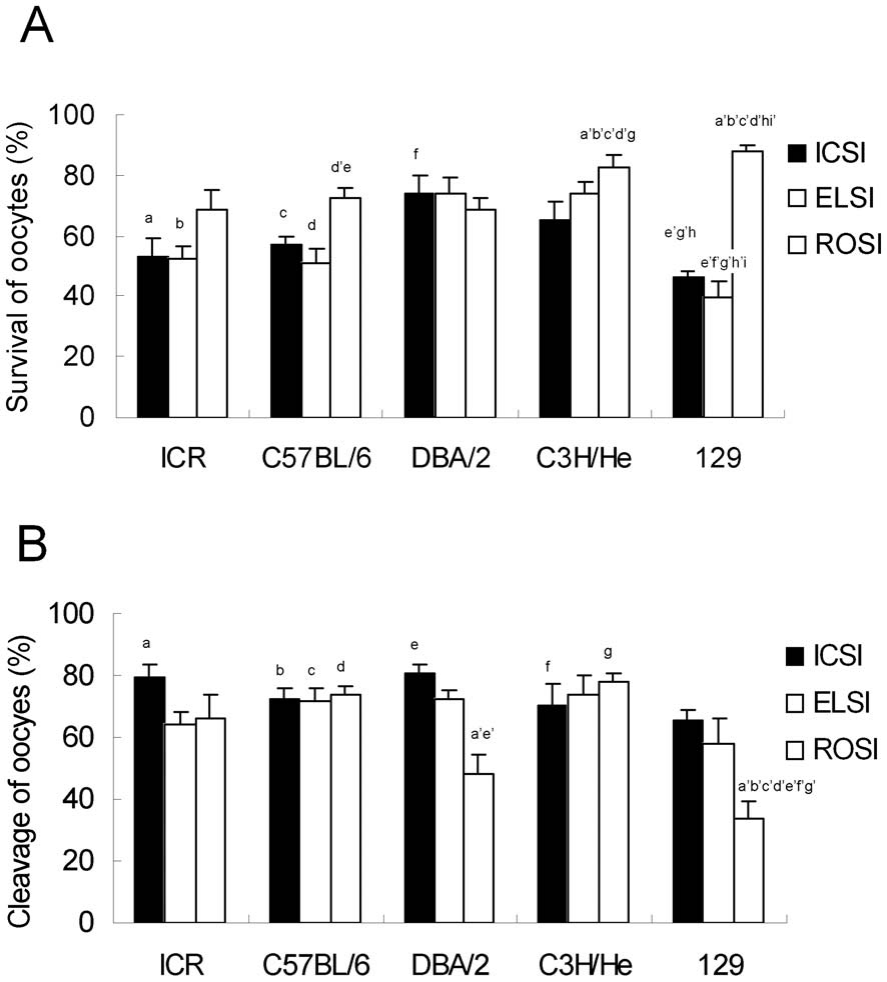
Effects of genotype and male germ cell type on oocyte survival (A) and cleavage to the 2-cell stage (B) following microinsemination. Data from fresh and frozen germ cells are combined and are expressed as the mean ± SEM. The combinations of characters from a-a′ to i-i′ indicate significant statistical differences (*P*<0.01) analyzed by multiple comparisons using the Tukey–Kramer procedure.

The in vitro and in vivo developmental outcomes of oocytes or embryos following microinsemination under different experimental conditions are summarized in Table S1. The best overall efficiency was obtained with frozen-thawed DBA/2 elongated spermatids (23.4%) while the lowest efficiency was observed for frozen–thawed 129 strain round spermatids (0.3%). Each developmental parameter was analyzed by three-way ANOVA for the presence or absence of statistically significant effects (Table 1). Genotype had significant effects on oocyte survival, cleavage, and birth rates, whereas the type of male germ cells had significant effects on all developmental parameters, including the overall efficiency calculated as the proportion of offspring produced per oocytes injected. By contrast, freeze–thawing treatment of male germ cells did not significantly affect any of the parameters observed. The presence or absence of significant interactions on the developmental parameters by two or three factors is shown in Table 1. For the oocyte survival and cleavage rates, there were significant interactions between the genotype and cell type, indicating that these parameters were determined by the combination of these two factors. We then applied multiple comparisons by merging the fresh and frozen groups for each of the 15 groups (5 genotypes ×3 cell types) (Table 1). There were 16 and 9 combinations of groups with significant differences for the oocyte survival and cleavage rates, respectively (Figure 1). For the implantation rate, birth rate, and overall efficiency, there were no interactions between factors so we separately applied multiple comparisons for each factor. The results revealed that spermatozoa and elongated spermatids gave better implantation and birth rates, and better overall efficiency than did round spermatids (Table 1).

**Table 1:** Probabilities (P-values) of main effects on each developmental parameter and their interactions.

		Oocyte survival^a^	Cleavage to 2-cells^a^	Implantation^b^	Birth^b^	Overall efficiency^b^
Main effect	Strain (Genotype)	**0.000**	**0.000**	0.054	0.005***	0.140
	Cell type	**0.000**	**0.000**	**0.000** (RoS < EpS, ElS)**	**0.000** (RoS < EpS, ElS)**	**0.000** (RoS < EpS, ElS)**
	Freezing	0.026	0.672	0.874	0.826	0.379
Interaction	Two factors (strain×cell type)	0.001*	0.001*	0.550	0.112	0.106
	Two factors (cell type×freezing)	0.410	0.167	0.218	0.136	0.063
	Two factors (strain×freezing)	0.227	0.336	0.964	0.488	0.315
	Three factors	0.145	0.967	0.035	0.028	0.035

Results were obtained by three-way ANOVA analysis. A probability of P<0.01 was considered significant (boldface).

a Oocyte survival and Cleavage to 2-cells: As there was an interaction between the strain and the cell type (single asterisks), the post hoc mutli comparisons were undertaken based on the groups of the [strain×cell type] combinations. For results, see Figure 1A and 1B, respectively.

b Implantation, Birth, and Overall efficiency: As there was no interaction between factors, post hoc multi comparisons were undertaken for individual factors and the results were indicated within the parenthesis (double asterisks). Although the birth rate was affected by the strain (triple asterisks), multi comparisons showed any significant differences between strains (P>0.01).

EpS, epididymal sperm; ElS, elongated spermatid; RoS; Round spermatid.

**Figure 2:**
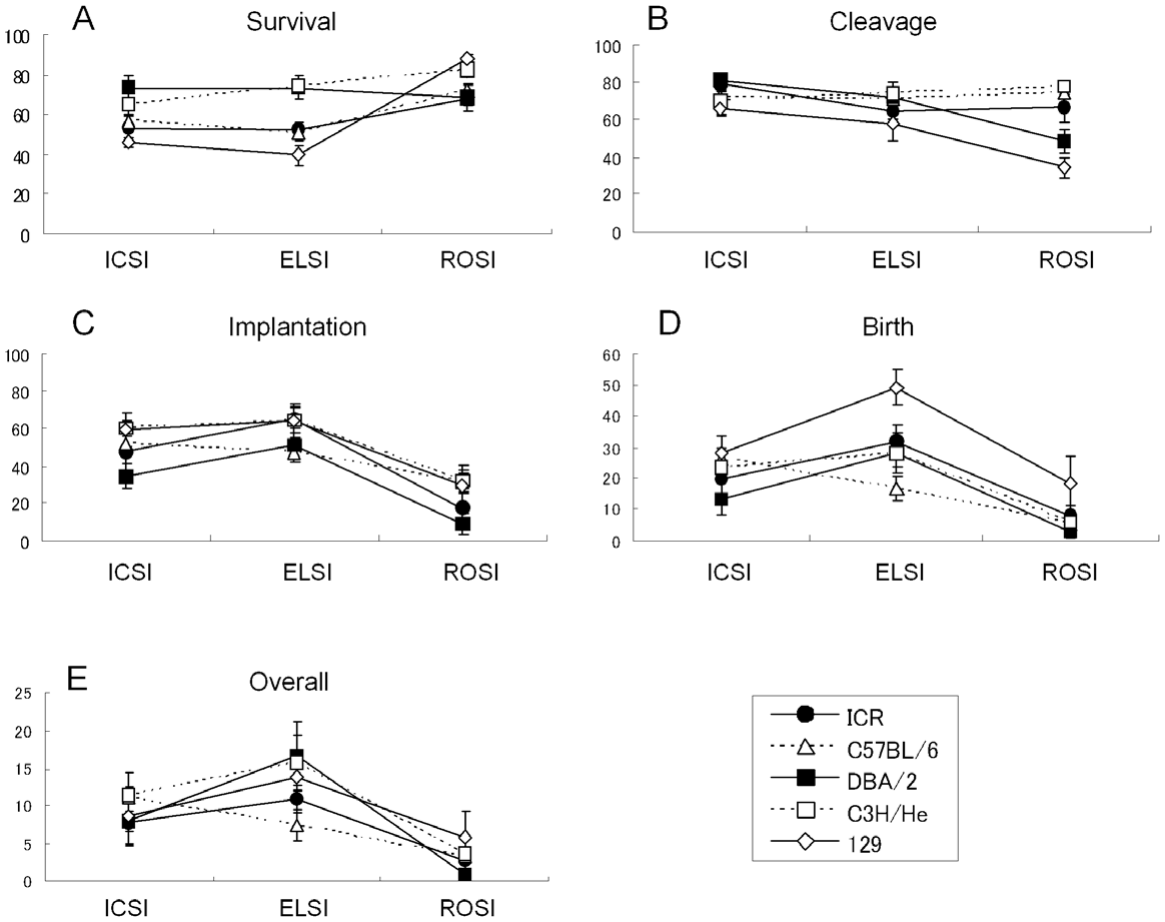
The developmental parameters of oocytes following ICSI, ELSI, or ROSI in five strains of mice. Data from fresh and frozen germ cells are combined and are expressed as the mean ± SEM.

We found that freeze–thawing of male germ cells did not have any significant effects or interactions with other factors in any of the parameters. Therefore, to demonstrate graphically the tendencies of genotype or cell type effects, we constructed interaction plots using these remaining two factors (Figure 2). For oocyte survival, the DBA/2 and C3H/He strains were superior to other strains and more oocytes survived after ROSI than after ICSI or ELSI (Figure 2A). For the cleavage rates, the efficiency was lowest for ROSI in the 129 strain (Figure 2B). For implantation, birth rates, and overall efficiencies, ICSI and ELSI were generally better than ROSI (Figures 2C, 2D, and 2E), which was consistent with results of the *post hoc* multiple comparisons described above. These plots are helpful to see the tendencies in the presence of interactions, which do not allow us to perform multiple comparisons within any single factor. In this study, we did not find any statistical differences between ICSI and ELSI for any of the parameters, and this tendency is also clearly shown in the interaction plots (Figure 2).

In the ROSI group of the DBA/2 strain, we often encountered recipient females with no implantation sites (9/11). We suspected that the DBA/2 ROSI embryos were poor at stimulating the uterus for an implantation response, including endometrial decidualization. We then undertook additional experiments to see whether the pregnancy rates could be improved by increasing the number of embryos transferred per each oviduct, as reported for IVF-generated embryos of this strain [39]. When 15–17 embryos were transferred into each oviduct, all three recipients became pregnant and the implantation and birth rates were greatly improved, reaching 69% and 38%, respectively (Table 2).

**Table 2:** Improved *in vivo* development of DBA/2 ROSI embryos transferred into recipient oviducts at large numbers.

No. injected	No. survived (% per injected)		No. cleaved (% per survived)		No. transferred per recipient (left + right)*	No. implanted (% per transferred)		No. pups (% per transferred)	
133	96	(72)	86	(90)	30 (15+15)	22	(73)	14	(47)
					30 (15+15)	19	(63)	11	(37)
					17 (17+0)	12	(71)	4	(24)
Total					77	53	(69)	29	(38)

*Eighty-six 2-cell embryos were allocated to 3 recipient females for transfer. The numbers in parentheses indicate the numbers of embryos transferred into the left and right oviduct, respectively.

## Discussion

Microinsemination, or ICSI, is a technique to deliver male germ cells directly into oocytes, and is a very effective technique to fertilize oocytes consistently, even with immotile spermatozoa or immature spermatogenic cells [3], [4]. Although many researchers have conducted microinsemination experiments and produced a number of offspring in different species to date, only little information is available for the factors that can affect the outcome of microinsemination. This probably arises from the difficulty in obtaining a large set of data for precise statistical analyses. In the present study, we took advantage of mice as laboratory species and completed a series of 185 ICSI experiments, thus allowing us to perform multiple factorial ANOVA analysis. As far as we know, the present study is the first to provide comprehensive information on ICSI, determining the effects of genotype, male germ cell type, and their freezing treatment on the development of ICSI-generated embryos. Our statistical analysis also revealed the presence of interactions among these three factors.

The first parameter we tested was the survival rate of oocytes after microinjection. We found a tendency for higher survival rates of oocytes from DBA/2 or C3H/He mice than those of other strains (Fig. 1). In general, the highest survival of oocytes is obtained using F1 hybrid mice, especially the combination of a C57BL/6 mother and a DBA/2 or C3H/He father (B6D2F1 and B6C3F1, respectively). They have contributed to technical advances in mouse microinsemination [5], [38] and cloning by somatic cell nuclear transfer [40]. Perhaps, the physical properties of oocytes might be inherited from the paternal strain. Indeed, the appearance of oocytes (opaqueness and the amount of cytoplasmic granules) from B6D2F1 females is very similar to the DBA/2 strain, and that from B6C3F1 females is very similar to the C3H oocytes (Ogonuki and Ogura, unpublished).

There was a tendency for more oocytes to survive after ROSI than after ICSI or ELSI. This is because of the smaller size of the injection pipette for ROSI, which helps to minimize the damage to recipient oocytes (about 3.8 µm vs. 5.0 µm for sperm microinjection). Furthermore, it is known that the preactivation of oocytes for ROSI makes them more robust than intact oocytes [20], although we do not know why.

The second parameter we examined was the cleavage rate to the 2-cell stage following microinsemination. There were no apparent strain-specific differences in the cleavage rates as long as we used mature spermatozoa or elongated spermatids. By contrast, for round spermatids, preactivation of oocytes was necessary because these immature gametes have no oocyte-activating capacity [41]. Therefore, the cleavage rate after ROSI reflects the responsiveness of the oocytes to the artificial activation. In this context, we can conclude that 129 strain oocytes are very insensitive to activation stimulus because only about 30–35% were cleaved after ROSI. In our preliminary experiments, we have confirmed the difficulty in activating 129 strain oocytes by strontium: many of them arrested at metaphase II (35%, 6/17) or metaphase III (47%, 8/17), and only 18% (3/17) developed to 2-cells (unpublished). Although it was reported that C57BL/6 strain oocytes also often arrested at metaphase III after parthenogenetic development [42], ROSI-generated embryos in this study developed into 2-cell embryos at normal rates (45–89%).

The developmental efficiency after embryo transfer was significantly affected by male germ cell type. As there were no interactions between factors for implantation rate, birth rate, and overall efficiency, we undertook multiple comparisons for each factor (Table 1). The analyses revealed that spermatozoa and elongated spermatids produced better rates for these parameters than did round spermatids. This indicates that ROSI-generated embryos have some specific difficulties in postimplantation development compared with ICSI- or ELSI-generated embryos. By contrast, embryos produced by ICSI or ELSI were generally indistinguishable from each other for any of the parameters examined. These findings clearly suggest that male germ cells might acquire a definite capacity for supporting embryonic development at some time during nuclear condensation in spermiogenesis. This assumption is consistent with the findings by Ohta et al. [19], who reported that the transition from late round spermatids (spermatogenesis steps 7–8) to elongating spermatids (steps 9–10) is the key point for the acquisition of the developmental ability similar to that of mature spermatozoa. However, no nuclear or subcellular events involved in this transition were so far identified. Further information should be important for more efficient and safer ELSI/ROSI in humans as well as in mice because there is also an apparent gap between the efficiencies of ELSI and ROSI in IVF clinics [43].

The genome of the mature spermatozoa is very stable to physical and chemical damage because of protection by nuclear protamines, which fold the DNA into a very compact structure [44]. By contrast, the nucleus of round spermatids still contains histones and is thought to be more sensitive to freezing and thawing damage than the nucleus of mature spermatozoa. Therefore, we first thought that the birth rates of embryos derived from frozen round spermatids would be lower than those from fresh round spermatids. However, this was not the case except for the 129 strain (35.8% vs. 0.7%), and factorial ANOVA analysis did not indicate any statistical differences between the fresh and freeze–thawing groups.

A remarkably low postimplantation development of ROSI-generated embryos was observed in the DBA/2 strain. This was probably caused by a failure in the uterine decidual reaction as evidenced by a complete lack of implantation scars in the uteri of some recipient females. Implantation is established by finely organized interactions between the embryo and the recipient mother, and it involves both the uterine microenvironment and the systemic endocrine milieu. As the mouse is a multiparous animal, the implanting embryos should cumulatively stimulate the uterus to induce the implantation reaction. Probably, the stimulus from each DBA/2 ROSI embryo is weaker than that of other strains, because we could overcome this implantation problem by increasing the number of embryos transferred into each female (Table 2). This indicates that the majority of DBA/2 strain ROSI-generated embryos potentially possessed the ability to complete postimplantation development, but the magnitude of their implantation signal(s) was less than the threshold needed.

The mouse is the most commonly used laboratory species because of the availability of large amounts of information on its genetics and biology. Another important advantage in the use of mice is that we can select the most appropriate type from the large stock of strains, including inbred, hybrid, outbred (closed-colony) strains according to the particular research purpose. These mouse strains might provide important backgrounds for generating gene-modified strains, such as transgenic or knockout mice, because the phenotypic effects of the modified genes can vary with their genetic background [45], [46]. However, only one founder or “last-of-line” animals lead to risks of losing invaluable mutant strains. In such situations, ICSI may be the only technique to rescue the lineage. Furthermore, mouse spermatozoa are sometimes inadequately cryopreserved and cannot be used for conventional IVF. Mouse ICSI can also be used for evaluation of in vitro-manipulated gametes and in this case, the production of offspring is the ultimate confirmation of functional competence in gametes [3], [4], [23]. Thus, it is most likely that the needs for mouse ICSI will increase further. We expect that our findings will provide valuable information for the most efficient use of animals, time, and experimental resources in biomedical research involving mouse ICSI.

## Supporting Information

Table S1 Overall developmental data.
(0.11 MB DOC)

## References

[1] Uehara T, Yanagimachi R (1976). Microsurgical injection of spermatozoa into hamster eggs with subsequent transformation of sperm nuclei into male pronuclei.. Biol Reprod.

[2] Uehara T, Yanagimachi R (1977). Behavior of nuclei of testicular, caput and cauda epididymal spermatozoa injected into hamster eggs.. Biol Reprod.

[3] Yanagimachi R (2005). Intracytoplasmic injection of spermatozoa and spermatogenic cells: its biology and applications in humans and animals.. Reprod Biomed Online.

[4] Ogura A, Ogonuki N, Miki H, Inoue K (2005). Microinsemination and nuclear transfer using male germ cells.. Int Rev Cytol.

[5] Ogura A, Matsuda J, Yanagimachi R (1994). Birth of normal young after electrofusion of mouse oocytes with round spermatids.. Proc Natl Acad Sci U S A.

[6] Kimura Y, Yanagimachi R (1995). Mouse oocytes injected with testicular spermatozoa or round spermatids can develop into normal offspring.. Development.

[7] Kimura Y, Yanagimachi R (1995). Development of normal mice from oocytes injected with secondary spermatocyte nuclei.. Biol Reprod.

[8] Ogura A, Suzuki O, Tanemura K, Mochida K, Kobayashi Y (1998). Development of normal mice from metaphase I oocytes fertilized with primary spermatocytes.. Proc Natl Acad Sci U S A.

[9] Miki H, Lee J, Inoue K, Ogonuki N, Noguchi Y (2004). Microinsemination with first-wave round spermatids from immature male mice.. J Reprod Dev.

[10] Tanemura K, Wakayama T, Kuramoto K, Hayashi Y, Sato E (1997). Birth of normal young by microinsemination with frozen-thawed round spermatids collected from aged azoospermic mice.. Lab Anim Sci.

[11] Hirabayashi M, Kato M, Aoto T, Ueda M, Hochi S (2002). Rescue of infertile transgenic rat lines by intracytoplasmic injection of cryopreserved round spermatids.. Mol Reprod Dev.

[12] Ogonuki N, Mochida K, Inoue K, Matsuda J, Yamamoto Y (2003). Fertilization of oocytes and birth of normal pups following intracytoplasmic injection with spermatids in mastomys (*Praomys coucha*).. Biol Reprod.

[13] Ogonuki N, Inoue K, Miki H, Mochida K, Hatori M (2005). Differential development of rabbit embryos following microinsemination with sperm and spermatids.. Mol Reprod Dev.

[14] Hirabayashi M, Kato M, Kitada K, Ohnami N, Hirao M (2009). Activation regimens for full-term development of rabbit oocytes injected with round spermatids.. Mol Reprod Dev.

[15] Tachibana M, Terada Y, Ogonuki N, Ugajin T, Ogura A (2009). Functional assessment of centrosomes of spermatozoa and spermatids microinjected into rabbit oocytes.. Mol Reprod Dev.

[16] Navara CS, Wu GJ, Simerly C, Schatten G (1995). Mammalian model systems for exploring cytoskeletal dynamics during fertilization.. Curr Top Dev Biol.

[17] Miki H, Inoue K, Ogonuki N, Mochida K, Nagashima H (2004). Cytoplasmic asters are required for progression past the first cell cycle in cloned mouse embryos.. Biol Reprod.

[18] Kishigami S, Van Thuan N, Hikichi T, Ohta H, Wakayama S (2006). Epigenetic abnormalities of the mouse paternal zygotic genome associated with microinsemination of round spermatids.. Dev Biol.

[19] Ohta H, Sakaide Y, Wakayama T (2009). Functional analysis of male mouse haploid germ cells of various differentiation stages: early and late round spermatids are functionally equivalent in producing progeny.. Biol Reprod.

[20] Miki H, Hirose M, Ogonuki N, Inoue K, Kezuka F (2009). Efficient production of androgenetic embryos by round spermatid injection.. Genesis.

[21] Shinmen A, Honda A, Ohkawa M, Hirose M, Ogonuki N (2007). Efficient production of intersubspecific hybrid mice and embryonic stem cells by intracytoplasmic sperm injection.. Mol Reprod Dev.

[22] Ogonuki N, Inoue K, Hirose M, Miura I, Mochida K (2009). A high-speed congenic strategy using first-wave male germ cells.. PLoS One.

[23] Kanatsu-Shinohara M, Shinohara T (2007). Culture and genetic modification of mouse germline stem cells.. Ann N Y Acad Sci.

[24] Byers SL, Payson SJ, Taft RA (2006). Performance of ten inbred mouse strains following assisted reproductive technologies (ARTs).. Theriogenology.

[25] Suzuki O, Asano T, Yamamoto Y, Takano K, Koura M (1996). Development in vitro of preimplantation embryos from 55 mouse strains.. Reprod Fertil Dev.

[26] Spearow JL (1988). Major genes control hormone-induced ovulation rate in mice.. J Reprod Fertil.

[27] Kawai Y, Hata T, Suzuki O, Matsuda J (2006). The relationship between sperm morphology and in vitro fertilization ability in mice.. J Reprod Dev.

[28] Dagnaes Hansen F, Hau J (1988). Quick freezing of mouse embryos: freezing of inbred strains and 2- and 4-cell embryos by vitrification.. In Vivo.

[29] Endoh K, Mochida K, Ogonuki N, Ohkawa M, Shinmen A (2007). The developmental ability of vitrified oocytes from different mouse strains assessed by parthenogenetic activation and intracytoplasmic sperm injection.. J Reprod Dev.

[30] Szczygiel MA, Kusakabe H, Yanagimachi R, Whittingham DG (2002). Intracytoplasmic sperm injection is more efficient than in vitro fertilization for generating mouse embryos from cryopreserved spermatozoa.. Biol Reprod.

[31] Ogonuki N, Mochida K, Miki H, Inoue K, Fray M (2006). Spermatozoa and spermatids retrieved from frozen reproductive organs or frozen whole bodies of male mice can produce normal offspring.. Proc Natl Acad Sci U S A.

[32] Kai M, Irie M, Okutsu T, Inoue K, Ogonuki N (2004). The novel dominant mutation *Dspd* leads to a severe spermiogenesis defect in mice.. Biol Reprod.

[33] Kawase Y, Iwata T, Toyoda Y, Wakayama T, Yanagimachi R (2001). Comparison of intracytoplasmic sperm injection for inbred and hybrid mice.. Mol Reprod Dev.

[34] Ohta H, Sakaide Y, Wakayama T (2009). Age- and substrain-dependent sperm abnormalities in BALB/c mice and functional assessment of abnormal sperm by ICSI.. Hum Reprod.

[35] Chatot CL, Ziomek CA, Bavister BD, Lewis JL, Torres I (1989). An improved culture medium supports development of random-bred 1-cell mouse embryos *in vitro*.. J Reprod Fertil.

[36] Ogura A, Yanagimachi R (1993). Round spermatid nuclei injected into hamster oocytes from pronuclei and participate in syngamy.. Biol Reprod.

[37] Ogura A, Matsuda J, Asano T, Suzuki O, Yanagimachi R (1996). Mouse oocytes injected with cryopreserved round spermatids can develop into normal offspring.. J Assist Reprod Genet.

[38] Kimura Y, Yanagimachi R (1995). Intracytoplasmic sperm injection in the mouse.. Biol Reprod.

[39] Kaneda H, Taguma K, Suzuki C, Ozaki A, Nakamura C (2007). An optimal embryo transfer condition for the effective production of DBA/2J mice.. Exp Anim.

[40] Wakayama T, Perry AC, Zuccotti M, Johnson KR, Yanagimachi R (1998). Full-term development of mice from enucleated oocytes injected with cumulus cell nuclei.. Nature.

[41] Kimura Y, Yanagimachi R, Kuretake S, Bortkiewicz H, Perry AC (1998). Analysis of mouse oocyte activation suggests the involvement of sperm perinuclear material.. Biol Reprod.

[42] Ibanez E, Albertini DF, Overstrom EW (2005). Effect of genetic background and activating stimulus on the timing of meiotic cell cycle progression in parthenogenetically activated mouse oocytes.. Reproduction.

[43] Al-Hasani S, Ludwig M, Palermo I, Küpker W, Sandmann J (1999). Intracytoplasmic injection of round and elongated spermatids from azoospermic patients: results and review.. Hum Reprod.

[44] Ward WS (2010). Function of sperm chromatin structural elements in fertilization and development.. Mol Hum Reprod.

[45] Glaser S, Anastassiadis K, Stewart AF (2005). Current issues in mouse genome engineering.. Nat Genet.

[46] Eisener-Dorman AF, Lawrence DA, Bolivar VJ (2009). Cautionary insights on knockout mouse studies: the gene or not the gene?. Brain Behav Immun.

