# Supplementary material for: The Effect on Intracytoplasmic Sperm Injection Outcome of Genotype, Male Germ Cell Stage and Freeze-Thawing in Mice
Source: PLoS One. 2010 Jun 11;5(6):e11062. doi: 10.1371/journal.pone.0011062 (PMC2884038; doi:10.1371/journal.pone.0011062)
Supplement: Table S1 — Overall developmental data. (0.11 MB DOC) [file pone.0011062.s001.doc]

| **Table S1.** Development *in vitro* and *in vivo* of embryos generated by microinsemination with different genotypes, male germ cell types, and their fresh/frozen condition. | | | | | | | | | | | | | | | | | | | | |
| --- | --- | --- | --- | --- | --- | --- | --- | --- | --- | --- | --- | --- | --- | --- | --- | --- | --- | --- | --- | --- |
| Genotype | Male germ cell type | Fresh or frozen | No. injected | % survived±SE (per injected) | | | % cleaved±SE (per survived) | | | No. transferred | % implanted±SE (per transferred) | | | No. of pups | % pups±SE (per transferred) | | | % overall efficiency±SE* | | |
|
| ICR | Sperm | Fresh | 275 | 47.2 | ± | 8.6 | 80.2 | ± | 5.3 | 93 | 48.3 | ± | 28.9 | 25 | 22.5 | ± | 13.0 | 9.3 | ± | 7.1 |
|  |  | Frozen | 240 | 59.0 | ± | 9.2 | 77.7 | ± | 6.9 | 44 | 46.9 | ± | 13.7 | 7 | 17.9 | ± | 4.1 | 6.8 | ± | 1.6 |
|  | Elongated spermatid | Fresh | 347 | 48.2 | ± | 4.2 | 60.7 | ± | 4.8 | 75 | 72.7 | ± | 11.4 | 28 | 34.8 | ± | 6.9 | 10.6 | ± | 2.6 |
|  |  | Frozen | 309 | 58.2 | ± | 5.6 | 68.3 | ± | 7.2 | 100 | 55.5 | ± | 1.3 | 25 | 28.6 | ± | 7.6 | 11.3 | ± | 3.4 |
|  | Round spermatid | Fresh | 144 | 77.2 | ± | 2.9 | 68.9 | ± | 11.5 | 88 | 10.1 | ± | 6.4 | 4 | 3.9 | ± | 2.3 | 1.5 | ± | 1.1 |
|  |  | Frozen | 168 | 53.0 | ± | 15.4 | 61.8 | ± | 5.3 | 51 | 27.7 | ± | 14.0 | 7 | 12.9 | ± | 7.2 | 4.3 | ± | 2.1 |
| C57BL/6 | Sperm | Fresh | 742 | 49.9 | ± | 2.7 | 78.9 | ± | 2.1 | 257 | 53.8 | ± | 6.2 | 70 | 29.9 | ± | 4.5 | 12.1 | ± | 2.1 |
|  |  | Frozen | 699 | 64.4 | ± | 4.8 | 65.2 | ± | 6.6 | 316 | 49.3 | ± | 7.4 | 73 | 22.7 | ± | 4.7 | 9.9 | ± | 2.5 |
|  | Elongated spermatid | Fresh | 772 | 44.4 | ± | 5.9 | 71.7 | ± | 5.0 | 200 | 43.4 | ± | 8.8 | 25 | 13.1 | ± | 5.7 | 5.3 | ± | 2.8 |
|  |  | Frozen | 342 | 61.7 | ± | 5.0 | 71.5 | ± | 6.8 | 138 | 54.9 | ± | 6.9 | 34 | 23.1 | ± | 4.7 | 10.9 | ± | 2.7 |
|  | Round spermatid | Fresh | 603 | 67.8 | ± | 5.1 | 75.0 | ± | 3.4 | 287 | 37.8 | ± | 7.3 | 21 | 5.9 | ± | 2.2 | 3.6 | ± | 1.5 |
|  |  | Frozen | 522 | 78.2 | ± | 4.4 | 72.8 | ± | 3.6 | 278 | 24.0 | ± | 6.4 | 16 | 4.9 | ± | 1.9 | 2.8 | ± | 1.0 |
| DBA/2 | Sperm | Fresh | 627 | 72.5 | ± | 7.8 | 84.9 | ± | 3.1 | 378 | 34.0 | ± | 8.5 | 63 | 17.6 | ± | 7.2 | 10.5 | ± | 4.4 |
|  |  | Frozen | 241 | 73.1 | ± | 7.3 | 84.8 | ± | 5.2 | 147 | 44.9 | ± | 10.6 | 29 | 22.5 | ± | 5.3 | 13.1 | ± | 1.3 |
|  | Elongated spermatid | Fresh | 284 | 65.7 | ± | 9.3 | 71.0 | ± | 5.7 | 133 | 38.7 | ± | 15.2 | 26 | 17.7 | ± | 7.8 | 9.8 | ± | 6.1 |
|  |  | Frozen | 233 | 81.8 | ± | 3.4 | 73.5 | ± | 3.6 | 140 | 63.4 | ± | 7.7 | 54 | 38.7 | ± | 6.1 | 23.4 | ± | 4.2 |
|  | Round spermatid | Fresh | 278 | 62.5 | ± | 6.5 | 41.4 | ± | 11.5 | 55 | 15.6 | ± | 15.6 | 2 | 4.4 | ± | 4.4 | 0.8 | ± | 0.8 |
|  |  | Frozen | 385 | 74.7 | ± | 3.5 | 54.5 | ± | 5.2 | 152 | 5.7 | ± | 5.7 | 5 | 2.2 | ± | 2.2 | 0.9 | ± | 0.9 |
| C3H/He | Sperm | Fresh | 248 | 69.0 | ± | 9.1 | 80.0 | ± | 8.5 | 135 | 61.2 | ± | 5.1 | 42 | 32.3 | ± | 5.2 | 19.0 | ± | 3.5 |
|  |  | Frozen | 338 | 61.9 | ± | 7.5 | 61.5 | ± | 10.0 | 130 | 59.1 | ± | 7.3 | 22 | 14.9 | ± | 2.1 | 3.9 | ± | 1.1 |
|  | Elongated spermatid | Fresh | 227 | 77.0 | ± | 5.8 | 75.0 | ± | 5.6 | 97 | 77.7 | ± | 11.5 | 31 | 29.2 | ± | 10.7 | 16.8 | ± | 7.1 |
|  |  | Frozen | 153 | 69.7 | ± | 4.1 | 72.4 | ± | 13.6 | 81 | 51.3 | ± | 9.5 | 23 | 27.4 | ± | 3.1 | 14.6 | ± | 4.2 |
|  | Round spermatid | Fresh | 374 | 78.8 | ± | 5.2 | 79.9 | ± | 4.3 | 217 | 27.0 | ± | 5.8 | 11 | 5.2 | ± | 1.3 | 3.1 | ± | 0.8 |
|  |  | Frozen | 238 | 91.1 | ± | 4.9 | 72.9 | ± | 2.5 | 145 | 44.4 | ± | 9.7 | 12 | 7.4 | ± | 2.3 | 4.8 | ± | 1.3 |
| 129 | Sperm | Fresh | 237 | 36.7 | ± | 2.5 | 68.4 | ± | 6.3 | 40 | 37.5 | ± | 19.1 | 7 | 22.9 | ± | 14.6 | 5.7 | ± | 4.4 |
|  |  | Frozen | 396 | 50.7 | ± | 2.1 | 64.5 | ± | 4.2 | 96 | 69.1 | ± | 8.6 | 31 | 30.4 | ± | 5.5 | 10.1 | ± | 2.4 |
|  | Elongated spermatid | Fresh | 219 | 30.6 | ± | 4.7 | 48.2 | ± | 12.7 | 30 | 59.3 | ± | 13.4 | 13 | 42.6 | ± | 10.3 | 7.4 | ± | 1.1 |
|  |  | Frozen | 174 | 51.6 | ± | 6.2 | 70.5 | ± | 6.1 | 60 | 69.2 | ± | 6.4 | 33 | 55.7 | ± | 3.4 | 20.3 | ± | 3.5 |
|  | Round spermatid | Fresh | 204 | 91.3 | ± | 1.8 | 32.4 | ± | 4.9 | 51 | 50.5 | ± | 9.9 | 20 | 35.8 | ± | 10.8 | 11.5 | ± | 5.2 |
|  |  | Frozen | 317 | 85.1 | ± | 1.1 | 35.6 | ± | 10.1 | 93 | 8.1 | ± | 8.1 | 1 | 0.7 | ± | 0.7 | 0.3 | ± | 0.3 |

Oocytes were injected with male germ cells from the same strain.

Experiments were replicated at least three times for each group.

*Calculated by (% survived) x (% cleaved) x (% pups)
